# *De novo KCNB1* mutations in infantile epilepsy inhibit repetitive neuronal firing

**DOI:** 10.1038/srep15199

**Published:** 2015-10-19

**Authors:** Hirotomo Saitsu, Tenpei Akita, Jun Tohyama, Hadassa Goldberg-Stern, Yu Kobayashi, Roni Cohen, Mitsuhiro Kato, Chihiro Ohba, Satoko Miyatake, Yoshinori Tsurusaki, Mitsuko Nakashima, Noriko Miyake, Atsuo Fukuda, Naomichi Matsumoto

**Affiliations:** 1 Department of Human Genetics, Yokohama City University Graduate School of Medicine, Yokohama, Japan; 2 Department of Neurophysiology, Hamamatsu University School of Medicine, Hamamatsu, Japan; 3 Department of Pediatrics, Epilepsy Center, Nishi-Niigata Chuo National Hospital, Niigata, Japan; 4 Epilepsy Center, Schneider’s Children Medical Center, Petah Tiqwa, Israel; 5 Department of Pediatrics, Yamagata University Faculty of Medicine, Yamagata, Japan

## Abstract

The voltage-gated Kv2.1 potassium channel encoded by *KCNB1* produces the major delayed rectifier potassium current in pyramidal neurons. Recently, *de novo* heterozygous missense *KCNB1* mutations have been identified in three patients with epileptic encephalopathy and a patient with neurodevelopmental disorder. However, the frequency of *KCNB1* mutations in infantile epileptic patients and their effects on neuronal activity are yet unknown. We searched whole exome sequencing data of a total of 437 patients with infantile epilepsy, and found novel *de novo* heterozygous missense *KCNB1* mutations in two patients showing psychomotor developmental delay and severe infantile generalized seizures with high-amplitude spike-and-wave electroencephalogram discharges. The mutation located in the channel voltage sensor (p.R306C) disrupted sensitivity and cooperativity of the sensor, while the mutation in the channel pore domain (p.G401R) selectively abolished endogenous Kv2 currents in transfected pyramidal neurons, indicating a dominant-negative effect. Both mutants inhibited repetitive neuronal firing through preventing production of deep interspike voltages. Thus *KCNB1* mutations can be a rare genetic cause of infantile epilepsy, and insufficient firing of pyramidal neurons would disturb both development and stability of neuronal circuits, leading to the disease phenotypes.

Voltage-gated K^+^ channels (Kv) play important roles in regulating electrical excitability in neurons^1^. They typically consist of tetrameric α subunits, with each subunit having six membrane-spanning α-helices (S1–S6). S1–S4 and S5–S6 segments form voltage-sensing and pore domains of the channel, respectively^2^. There are 12 Kv subfamilies (Kv1 to Kv12) in humans, which contribute to diverse aspects of neuronal signaling^1^,^3^. Heterozygous mutations in Kv are involved in epileptic disorders, for example *KCNA2* (Kv1.2), *KCNC1* (Kv3.1), *KCNQ2* (Kv7.2), *KCNQ3* (Kv7.3), and *KCNH1* (Kv10.1)^4^,^5^,^6^,^7^,^8^,^9^,^10^.

Kv2 includes Kv2.1 and Kv2.2, encoded by *KCNB1* at 20q13.13 and *KCNB2* at 8q13.3, respectively. Kv2.1/KCNB1 is expressed in both excitatory and inhibitory neurons throughout the brain^11^,^12^,^13^, and produces the major delayed rectifier K^+^ current in hippocampal and cortical pyramidal neurons^14^. Kv2.1 expression can be modulated by neuronal activity via phosphorylation^15^, which may control homeostatic changes in neuronal excitability^14^.

Recently, *de novo* heterozygous *KCNB1* mutations were reported in three patients with epileptic encephalopathy^16^,^17^,^18^ and a patient with neurodevelopmental disorder^19^. Functional analysis of three missense mutations (p.S347R, p.T374I and p.G379R) in the pore domain showed loss of ion selectivity and gain of depolarizing inward cation conductance in CHO-K1 cells^16^, suggesting that the mutations may cause depolarized resting membrane potential and impaired membrane repolarization, which would lead to increased excitability of neurons. However, actual mutational effects on neuronal activity, which must be influenced by various properties of ion channels and other molecules, and frequency of *KCNB1* mutations in epileptic patients, remains to be elucidated.

In this study, we identified two patients with novel *de novo* heterozygous *KCNB1* mutations (p.R306C and p.G401R) among 437 patients with infantile epilepsy. These mutations were located in the voltage-sensing and pore domains, respectively. Extensive functional examination using the whole-cell patch-clamp technique in Neuro2a cells and primary cortical neurons showed that these Kv2.1 mutants, with different mechanisms from the previously reported ones, inhibit production of sufficiently deep interspike voltages, thereby inhibiting repetitive action potential (AP) firing to different degrees in cortical neurons. Our study highlighted the importance of Kv2.1 for controlling neuronal activity in humans.

## Results

### Identification of *de novo KCNB1* mutations

We have been conducting whole exome sequencing (WES) analysis of inherited intractable disorders including infantile epilepsy as a national project since 2011. Our attention to *KCNB1* mutations was raised by finding a *de novo* heterozygous *KCNB1* mutation in patient 1 by trio-based WES, in which the mean read depth of protein-coding regions in RefSeq genes ranged from 138–161, with 94.7–95.6% of target coding sequences covered by 10 or more reads (Supplementary Table S1). In this trio analysis, we identified two *de novo* mutations and two candidate X-linked recessive variants (Supplementary Table S2). Among them, the *de novo* mutation in *KCNB1* (c.1201G>A (p.G401R)) was the most likely candidate because *KCNB1* encodes Kv2.1, which is the major delayed rectifier Kv that regulates cortical pyramidal neuron excitability^14^. This assumption was later justified by recent reports of three patients with *de novo KCNB1* mutations and epileptic encephalopathy^16^,^17^,^18^. Searching WES data of other 436 patients, we identified another candidate heterozygous *KCNB1* mutation (c.916C>T (p.R306C)) in patient 2, and confirmed it as *de novo* by Sanger sequencing. Two mutations were absent in dbSNP 137, our in-house 575 control exomes, and Exome Variant Server database (Supplementary Table S2).

**Figure 1:**
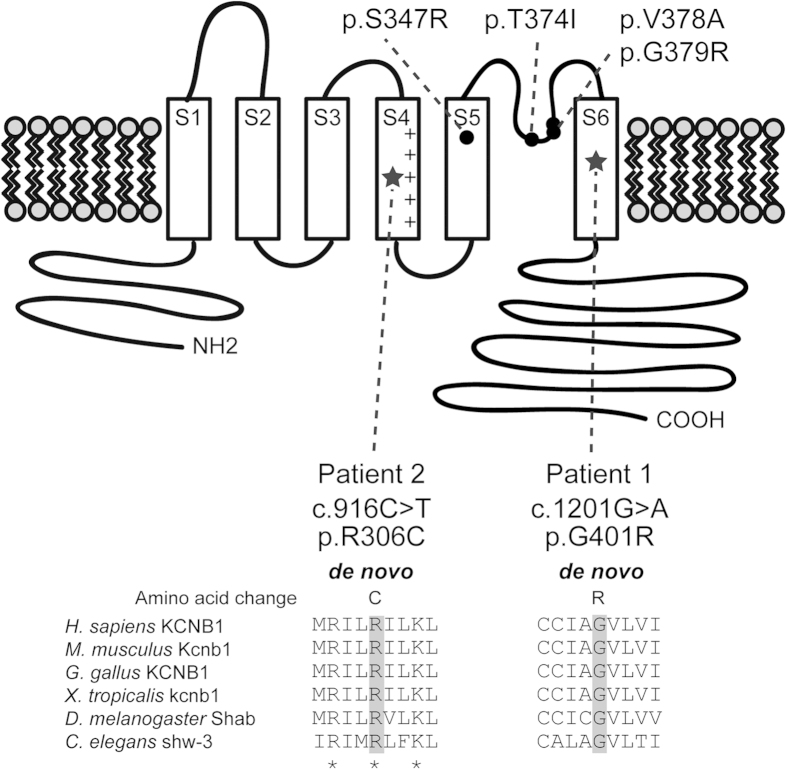
Location of *KCNB1* mutations on human Kv2.1 protein obtained from UniProt (Q14721, http://www.uniprot.org/uniprot/). Four previously reported mutations^16^,^17^,^18^,^19^ (black circles) and the two mutations identified in this study (stars) are shown. The p.R306C mutation occurs at a conserved positively charged residue (asterisk) in the S4 segment, which is important for voltage sensing^20^. The p.G401R mutation substitutes a highly conserved glycine residue in the S6 segment, which functions as a gating hinge^2^. Homologous sequences were aligned using the CLUSTALW web site (http://www.genome.jp/tools/clustalw/).

Locations of the two *KCNB1* mutations along with four previously reported mutations are illustrated in Fig. 1. The p.G401R mutation is located in S6 of the pore domain, in which the four reported mutations were also located. The p.R306C mutation is located in S4 of the voltage-sensing domain. Web-based prediction tools suggested that these two mutations could affect protein function (Supplementary Table S2). Interestingly, the two missense mutations occurred at evolutionarily conserved amino acids important for Kv gating (Fig. 1): R306 is one of positively charged residues (mainly arginines) in S4, responsible for voltage sensing^20^, and G401 in S6 functions as a gating hinge^2^. Therefore, p.R306C and p.G401R mutations are predicted to affect voltage sensing and pore gating of Kv2.1, respectively.

### Current properties of Kv2.1 mutants expressed in Neuro2a cells

**Figure 2:**
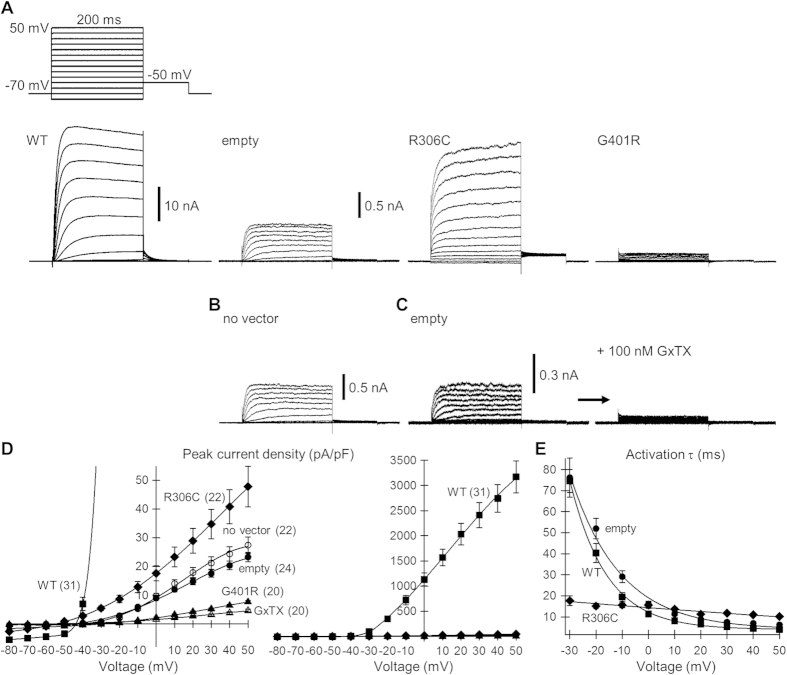
I–V relationships and activation τs of Kv currents in Kv2.1 mutant-transfected Neuro2a cells. (**A**) Representative currents in cells transfected with WT Kv2.1, empty vector, R306C and G401R mutants of Kv2.1. All currents were elicited with the voltage steps in 10 mV increments from the holding potential of −70 mV, as depicted above the WT trace. (**B**) Currents in untransfected cells (no vector). (**C**) Currents in empty were abolished by bath application of 100 nM GxTX. (**D**) I–V relationship of peak current densities during voltage steps. The relationship in WT (solid squares) is shown in the right panel on a larger scale because of its greater magnitude. The relationship in no vector (open circles) was not statistically different from that in empty (solid circles, *P* = 0.90 for current density at +50 mV), whereas R306C (solid diamonds), G401R (solid triangles), and GxTX (open triangles) were significantly different from empty at a current density of +50 mV (*P *= 0.025 vs R306C, *P *< 0.01 vs G401R and GxTX by Dunnett’s T3 test). The numbers of cells analyzed are indicated in parentheses. (**E**) Voltage dependence of activation τ. τs in empty and WT were determined by fitting the initial current rising phase from 20% of the peak rise to single exponentials. Currents in R306C were similarly fitted to double exponentials and the initial faster τs plotted. τs at −30 and −20 mV in R306C were smaller than in WT (*P* < 0.01 by Dunnett’s T3 test), whereas at +50 mV it was larger than in WT (*P* < 0.01 by REGW test).

To examine the mutational effect on Kv2.1 function, we expressed the Kv2.1 mutants in Neuro2a cells and performed voltage-clamp analysis of membrane currents. Neuro2a cells are reported to have no endogenous Kv conductance^21^,^22^, although a significant voltage-gated current was detected in another report^23^. In our culture and experimental conditions, small but significant voltage-gated outward currents were generated in both cells transfected with empty vector and untransfected cells (Fig. 2A–D, empty and no vector), confirming presence of endogenous voltage-gated currents. Currents were activated in response to voltage steps ≥ −30 mV, with slow activation kinetics in the negative voltage range and much faster kinetics in the positive voltage range (Fig. 2E), reminiscent of currents mediated by wild-type (WT) Kv2, as reported previously^24^,^25^,^26^. Indeed, application of guangxitoxin-1E (a specific Kv2 blocker; GxTX) abolished these currents (Fig. 2C,D). Thus, the endogenous currents were solely mediated by Kv2 in our experimental conditions.

**Figure 3:**
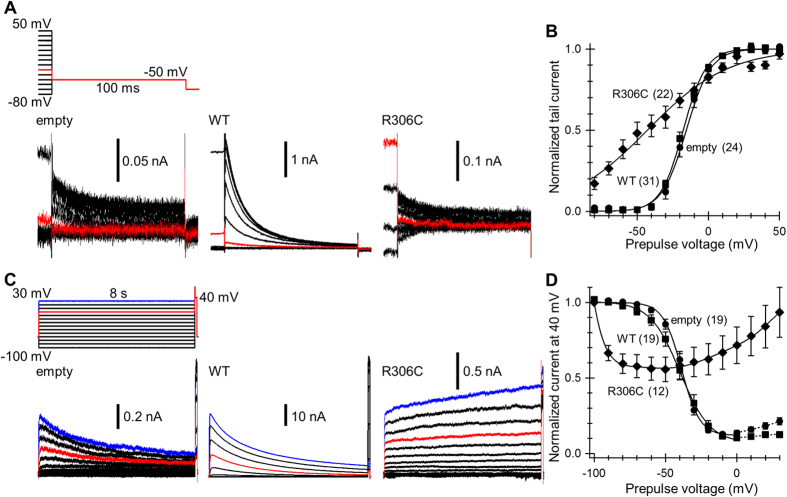
Voltage dependence of activation and inactivation of Kv currents in transfected Neuro2a cells. (**A**) Representative tail currents. Traces from the last 10 ms of 200 ms voltage steps are shown. Currents elicited by the step from –30 mV are highlighted in red. (**B**) Activation curves of endogenous Kv2 (empty; circles), WT (squares), and R306C (diamonds). V_h_ and V_s_ in empty (−15.6 ± 2.2 mV and 7.0 ± 0.7 mV, respectively) did not differ statistically from those in WT (V_h_ −17.4 ± 1.3 mV, *P* = 0.85; V_s_ 6.9 ± 0.2 mV, *P* = 1.00 by Dunnett’s T3 test). V_h_ and V_s_ in R306C were −43.3 ± 5.6 mV and 25.7 ± 2.5 mV, respectively. (C) Representative currents evoked by long voltage steps. Currents evoked by the steps to 0 mV and +30 mV are highlighted in red and blue, respectively. (D) Inactivation curves of endogenous Kv2 (circles), WT (squares), and R306C (diamonds). V_h_ and V_s_ in empty were −38.7 ± 1.3 mV and 5.7 ± 0.2 mV, respectively, and in WT were −39.9 ± 2.7 mV (*P* = 0.68) and 7.3 ± 0.3 mV (*P* < 0.01 by 2-tailed Student’s *t* test), respectively. In R306C, the current after the prepulse to +30 mV (0.93 ± 0.33) was significantly larger than that after the prepulse to −50 mV (0.56 ± 0.08, *P* < 0.01 by 2-tailed paired Student’s *t* test). The numbers of cells analyzed are indicated in parentheses.

Transfection of cells with WT Kv2.1 produced currents two orders of magnitude larger than endogenous currents (Fig. 2A,D). Voltage dependence of the time constant (τ) of current activation was similar to that of endogenous currents (Fig. 2E). Transfection with R306C mutant produced currents approximately two times larger than endogenous currents (Fig. 2A,D). However, current-voltage (I–V) dependence and activation kinetics differed from WT currents. Significant current responses were even detected at < −30 mV, and the typical slow activation kinetics in the negative voltage range were lost. Activation τs at the beginning of voltage steps between −30 and −20 mV were much smaller than those of WT currents (Fig. 2E). Nevertheless, voltage dependence of τ was so weak that the τ at +50 mV was significantly larger than the WT current (Fig. 2E). Moreover, especially in the positive voltage range, slow rises in current amplitude continued throughout the voltage steps after the initial rapid rising phase (Fig. 2A). The rises persisted even when the voltage step period was extended to 8 s (Fig. 3C), and the current response to a test voltage pulse after the voltage step still increased with increasing level of the voltage step (Fig. 3C,D). In WT and endogenous currents, the rise in amplitude stopped after the initial rising phase and was followed by slow current inactivation, particularly in the positive voltage range (Fig. 3C). Thus, these findings suggest that loss of one electric charge at R306 in the S4 voltage sensor strongly affects sensitivity and cooperativity of the sensor, thereby slowing channel opening and inactivation. Indeed, tail current analysis confirmed disruption of voltage sensor sensitivity and cooperativity in the R306C mutant (Fig. 3A,B). In stark contrast to the R306C mutant, transfection with G401R mutant nearly abolished endogenous currents, similar to GxTX (Fig. 2A,D). This suggests that the G401R mutant does not function as an ion channel and may exert a dominant-negative effect on WT channels.

### Characteristics of K^+^ currents in primary cortical neurons expressing Kv2.1 mutants

**Figure 4:**
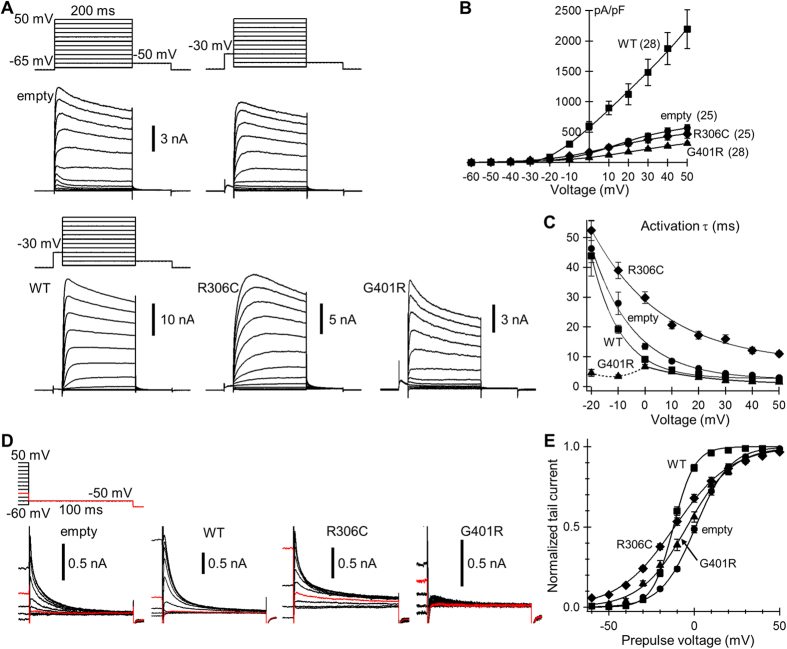
Voltage dependence of Kv activation in Kv2.1 mutant-transfected primary cortical neurons. (**A**) Representative Kv currents. Except the currents in the upper left, 25 ms prepulses to −30 mV were included to isolate I_A_. Note that the currents during the following voltage steps at –30 mV after the prepulses were noticeable only in R306C. (**B**) I–V relationships of peak currents. To include I_A_, prepulses were not applied. The current density at +50 mV in R306C (diamonds) did not differ from that in empty (circles, *P *= 0.83), whereas the density in G401R (triangles) and WT (squares) differed (*P *< 0.01). (**C**) Voltage dependence of τ. To exclude I_A_, prepulses were applied. τs were determined by fitting the initial current rising phase from 20% of the peak rise to single or double exponentials, depending on better fit. When currents were fitted to double exponentials, slower τs were plotted. τs at ≥ 0 mV in R306C were larger than in others (*P *< 0.01). τs at −20 and −10 mV in G401R (dotted line) were smaller than in others (*P *< 0.01). (**D**) Representative tail currents. The currents were elicited by a 100 ms test voltage step to −50 mV after 200 ms voltage steps ranging from –60 mV to +50 mV in 10 mV increments. Traces from the last 10 ms of 200 ms voltage steps are shown. Currents elicited by the step from –30 mV are highlighted in red. (**E**) Activation curves. V_h_ and V_s_ in empty (1.0 ± 1.0 mV and 9.7 ± 0.4 mV, respectively) differed from those in WT (V_h_ −11.9 ± 0.8 mV, *P *< 0.01; V_s_ 5.9 ± 0.4 mV, *P *< 0.01), R306C (V_h_ −11.1 ± 1.3 mV, *P *< 0.01; V_s_ 16.3 ± 1.0 mV, *P* < 0.01), and G401R (V_h_ −4.7 ± 1.6 mV, *P* < 0.05; V_s_ 12.9 ± 0.8 mV, *P* < 0.01). V_h_ in R306C did not differ from WT (*P* = 1.00), although V_s_ did (*P *< 0.01). All comparisons here were by Dunnett’s T3 test.

To investigate how Kv2.1 aberrations may cause epileptic phenotypes, we next analyzed the mutational effect on total K^+^ currents and firing activities in cultured cortical neurons. The K^+^ current in cortical neurons consists of multiple Kv components including Kv2. Applying voltage steps to neurons transfected with empty vector evoked endogenous Kv currents 20-times larger than in Neuro2a cells (Fig. 4A, empty, left panel, and 4B). The transient outward current component elicited at the beginning of voltage steps between −40 and 0 mV is the typical KCND/Kv4-mediated current, so called “I_A_”. This component can be separated from the total current by applying a 25 ms prepulse to −30 mV because of its fast activating and inactivating property at this voltage (Fig. 4A, empty, right panel). The slowly activating component elicited by voltage steps between −20 and 0 mV after I_A_ removal strongly implicates endogenous Kv2. Indeed, activation τs of the slowly activating component (Fig. 4C, empty) matched those in Neuro2a cells (Fig. 2E, empty). However, τs in the positive voltage range (e.g., 2.9 ± 0.3 ms at +50 mV, *n* = 25) were significantly smaller than in Neuro2a cells (6.2 ± 0.4 ms at +50 mV, *n* = 24, *P* < 0.01 by 2-tailed Student’s *t* test). Moreover, the rate of current inactivation in the positive voltage range (e.g., τ of 248.4 ± 37.6 ms at +30 mV) was clearly faster than in Neuro2a cells (3268.2 ± 172.0 ms). These findings presumably reflect faster activation and inactivation of other Kv types.

Transfection of neurons with WT Kv2.1 produced currents approximately four times larger than endogenous currents (Fig. 4A,B). After I_A_ removal, activation τs in the voltage range between −20 and 0 mV (Fig. 4C, WT) also matched those in Neuro2a cells (Fig. 2E, WT). However, activation and inactivation rates in the positive voltage range were faster than in Neuro2a cells, as were those of endogenous currents. Transfection with R306C mutant produced currents that were similar in amplitude to endogenous currents in the positive voltage range (Fig. 4B) but larger in the negative voltage range (Fig. 4A and Supplementary Fig. S1). Moreover, the activation time course after I_A_ removal was much slower than WT and endogenous currents, especially in the positive voltage range (Fig. 4A,C). Voltage dependence of the activation τ in R306C-transfected neurons was different from Neuro2a cells (Fig. 2E, R306C). The reason for the difference is discussed later. Transfection with G401R mutant produced significantly smaller peak currents (Fig. 4B) and faster rates of current inactivation (e.g., 92.7 ± 5.8 ms at +50 mV, *n* = 28) compared with endogenous currents (145.7 ± 12.9 ms at +50 mV, *n* = 25, *P* < 0.01 by 2-tailed Student’s *t* test; Fig. 4A). Moreover, the typical slow activation kinetics in the negative voltage range were completely lost (Fig. 4A,C). This can be explained by selective suppression of endogenous Kv2 by the dominant-negative action of the G401R mutant. Only fast activating and inactivating current components mediated by other Kv types contributed to the current response. This was also indicated by the tail currents after the voltage steps (Fig. 4D). In general, deactivation processes of fast activating Kv components (such as Kv1/KCNA and Kv3/KCNC) are too fast to be detected as tail currents. Thus, the currents observed here mainly reflect deactivation processes of slowly activating components like Kv2. The peak current density of tail currents in G401R-transfected neurons was greatly suppressed (4.8 ± 1.8 pA/pF, after +50 mV voltage steps) compared with endogenous currents (31.3 ± 4.2 pA/pF, *P* < 0.01 by Kruskal-Wallis test), although smaller but comparable current amplitudes were generated during the preceding voltage steps (Fig. 4A,B).

Tail currents in WT- and mutant-transfected neurons also indicate differences in voltage dependence of slowly activating components, including Kv2. The half-maximal activation voltage significantly shifted to the left in WT Kv2.1-transfected neurons compared with empty vector-transfected neurons (Fig. 4E). This was due to dominance of WT Kv2.1 with half-maximal activation of approximately −17 mV (Fig. 3B) in the slowly activating components. A marked increase in slope factor was apparent in R306C-transfected neurons (Fig. 4E), consistent with our finding in Neuro2a cells (Fig. 3B). This was reflected in significant tail currents even after voltage steps to −30 and −40 mV in R306C-transfected neurons only (Fig. 4D) and also in the larger currents during the preceding voltage steps in the voltage range less than –20 mV in R306C-transfected neurons, compared with those in others (Fig. 4A and Supplementary Fig. S1).

### Kv2.1 mutants inhibit repetitive AP firing

**Figure 5:**
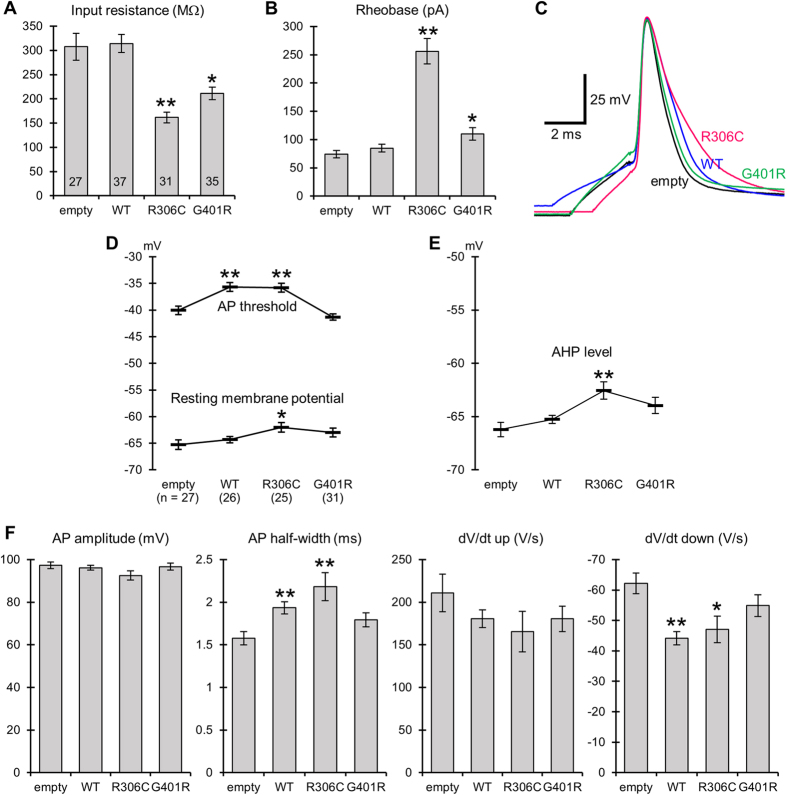
Passive membrane properties and characteristics of a single action potential spike evoked by a short 2–5 ms current pulse in Kv2.1 mutant-transfected primary cortical neurons. (**A**) Comparison of input resistance under resting conditions. *P < 0.05 compared with empty and R306C, and *P *< 0.01 compared with WT. **P < 0.01 compared with empty and WT, and *P *< 0.05 compared with G401R by Dunnett’s T3 test. (**B**) Comparison of rheobase. *P < 0.05 compared with empty, and *P *< 0.01 compared with R306C. **P < 0.01 compared with empty, WT, and G401R by Dunnett’s T3 test. The numbers of cells analyzed in (**A**,**B**) are indicated in the bars in (**A**). (**C**) Representative traces of single APs evoked by 2–5 ms current injections. Duration of current injection was adjusted, therefore injected currents did not affect spike upstrokes. Spike upstrokes were superimposed to highlight differences in speed of spike repolarization. (**D**) Resting membrane potentials and voltage thresholds for spike generation (AP threshold). *P < 0.05 compared with resting membrane potential in empty by REGW *F* test. **P < 0.01 compared with AP thresholds in empty and G401R by REGW *F* test. (**E**) Minimum voltage level of afterhyperpolarization (AHP level). **P < 0.01 compared with AHP level in empty, and *P *< 0.05 compared with WT by Dunnett’s T3 test. (**F**) Amplitude (AP amplitude), half-width (AP half-width), speed of spike upstrokes (dV/dt up) and downstrokes (dV/dt down) for a single action potential. *P < 0.05, **P < 0.01 compared with empty by Dunnett’s T3 test. There were no statistical differences in these spike parameters between WT and R306C. The number of cells analysed in (**D–F**) are indicated in the parentheses in (**D**).

Next, we examined changes in passive membrane properties and firing activities of transfected neurons under current-clamp conditions. We found notable changes in passive membrane properties of R306C-transfected neurons, specifically, large decreases in input resistance (Fig. 5A) and corresponding increases in the minimum stimulus current required for AP firing (rheobase) (Fig. 5B). These observations are due to a significant fraction of open R306C mutants below the threshold for AP firing (Fig. 4A and Supplementary Fig. S1), because of the corresponding large slope factor of voltage dependence (Figs 3B and 4E). To a lesser degree, similar changes were also observed in G401R-transfected neurons. The shape of a single AP evoked by a short (2–5 ms) current injection was varied in WT- and R306C-transfected neurons. The half-width of AP spike was prolonged, compared with empty vector-transfected neurons, owing to reduction in speed of the spike downstroke without change in the spike upstroke (Fig. 5C,F). There were no statistical differences in these spike parameters between WT- and R306C-transfected neurons. Other minor changes in passive membrane properties and in single spike parameters are listed in Fig. 5D–F.

**Figure 6:**
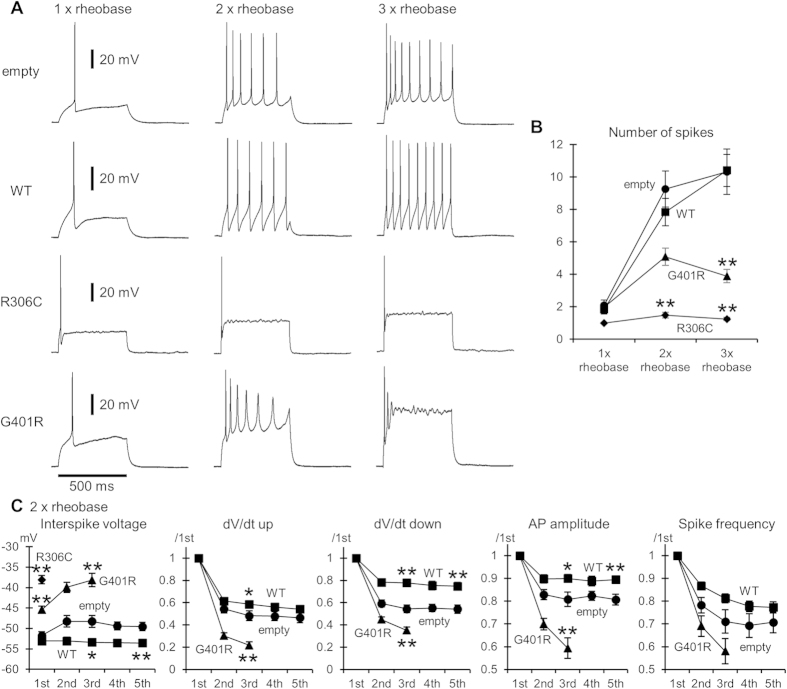
Repetitive firing properties of Kv2.1 mutant-transfected primary cortical neurons. (**A**) Representative traces of repetitive AP firing evoked by 500 ms current injections of one, two, or three times the rheobase amplitude. (**B**) Changes in the number of AP spikes generated during a 500 ms current injection with increasing current amplitude. **P < 0.01 compared with others by Kruskal-Wallis test. (**C**) Changes in the minimum interspike voltage level, maximum speed of spike upstrokes (dV/dt up) and downstrokes (dV/dt down), spike amplitude (AP amplitude), and instantaneous spike frequency (reciprocal of the interspike interval) during repetitive firing evoked by a 500 ms current injection of two times the rheobase. Parameters values of the initial five spikes and intervals in empty and WT, up to the third spike in G401R, and only the first interspike voltage in R306C were compared, because of differences in average spike number during injections, as shown in (**B**). Values of dV/dt up, dV/dt down, AP amplitude, and spike frequency were normalized to the first spike and frequency. *P < 0.05, **P < 0.01 compared with empty by Dunnett’s T3 test. The first interspike voltages in R306C and in G401R also differed significantly (*P *< 0.01). The numbers of cells analyzed in (B,C) are indicated in the bars in Fig. 5A.

The pattern of repetitive AP firing elicited by a long 500 ms current injection varied greatly between neurons transfected with different vectors. In empty vector-transfected neurons, the spike number during current injection increased up to around 10 with increasing strength of current injection (up to three times the rheobase) (Fig. 6A,B, empty). Similar increases in spike number were observed in WT-transfected neurons (Fig. 6A,B, WT). In stark contrast, in R306C-transfected neurons, increased current strength did not increase spike number: only 1 or 2 spikes were generated at the beginning of current injections in most cases (Fig. 6A,B). In G401R-transfected neurons, a current increase of two times the rheobase increased the spike number to around 5. However, spikes were likely dampened during injection, and a further current increase suppressed the repetitive firing (Fig. 6A,B).

To understand the basis for spike pattern differences, we further examined several spike parameters during repetitive firing induced by a two times rheobase current. In empty vector-transfected neurons, the minimum interspike voltage between the first and second spikes first increased by 2–3 mV in the next interval, and was then sustained during the following repetitive firing (Fig. 6C, interspike voltage). In WT-transfected neurons, the interspike voltage was sustained from the beginning, with the final level being lower than that in empty vector-transfected neurons. In R306C-transfected neurons, the initial interspike voltage was much higher than in empty- and WT-transfected neurons. The initial interspike voltage in G401R-transfected neurons was between that of empty- and R306C-transfected neurons, but the voltage rose progressively during repetitive firing. These differences in interspike voltage may affect the speed of spike upstroke in the following spikes, because recovery of Na^+^ channels from inactivation is hampered at high voltages. Indeed, the upstroke speed in the middle of repetitive firing was greatly suppressed in G401R-transfected neurons, whereas it was largely maintained in WT-transfected neurons (Fig. 6C, dV/dt up). The upstroke speed may in turn affect the subsequent spike downstrokes, amplitudes, and frequencies, because Kv opening depends greatly upon membrane depolarization caused by Na^+^ influx. In fact, all of these parameters followed the same trend as the spike upstroke (Fig. 6C). These findings confirm the important role of WT Kv2.1 in preserving low interspike voltages to maintain repetitive firing, as reported previously^26^,^27^. The novel Kv2.1 mutants inhibit production of sufficiently deep interspike voltages, thereby inhibiting repetitive firing to different degrees.

### Clinical features of patients with *KCNB1* mutations

**Figure 7:**
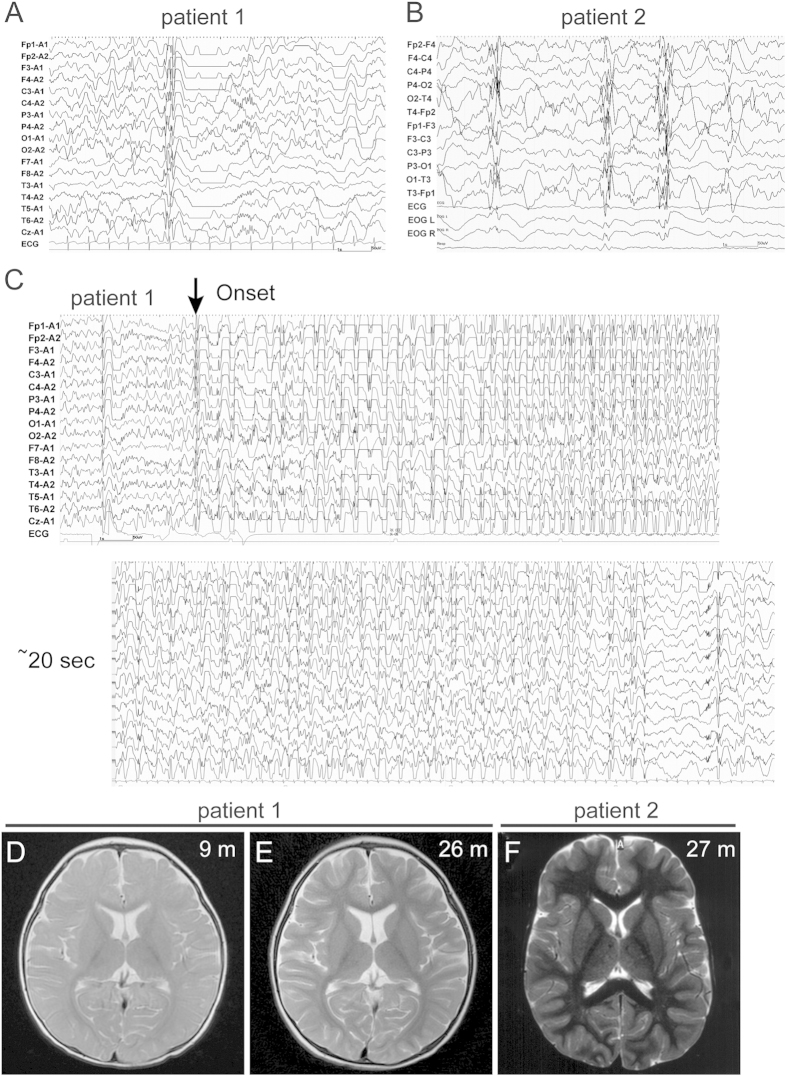
EEG and brain MRI features of patients with *KCNB1* mutations. (**A,B**) Interictal EEG tracing of patient 1 at 18 months of age, and patient 2 at 2 years and 11 months. (**A**) Diffuse irregular polyspikes and waves with intermittent focal spikes during the sleep state in patient 1. (**B**) Diffuse irregular slow spikes and waves, and polyspikes and waves during the sleep state in patient 2. (**C**) Ictal EEG tracing of patient 1 at 23 months showed runs of diffuse slow spike and wave discharges beginning at 2 Hz and accelerating to 3–3.5 Hz irregular spikes and waves, followed by a mixture of irregular spikes intermingled with arrhythmic slow activities that accompanied generalized clonic seizures. Seizures lasted approximately 50 seconds. (**D–E**) T2-weighted axial brain MRI of patient 1. (**D**) MRI at 9 months showed normal findings. (**E**) MRI at 2 years 2 months showed slight cortical atrophy especially in the frontal lobes. (**F**) T2-weighted axial brain MRI of patient 2 showed normal findings. m, months; y, years

The clinical features of two patients with *KCNB1* mutations are summarized in Table 1, along with three previously published patients^16^,^17^,^18^. In another patient^19^, detailed clinical information was unavailable from the literature. They initially showed developmental delay, and thereafter developed various infantile-onset seizures. Of note, generalized seizures with high-amplitude diffuse spike and wave electroencephalogram (EEG) discharges were commonly observed in two patients (Fig. 7A–C and Supplementary Fig. S2). Brain MRI showed normal findings, but patient 1 later showed progressive brain atrophy especially in the cortex (Fig. 7D–F). Case reports are available in the Supplementary Information.

## Discussion

In this study, we identified two novel *de novo KCNB1* mutations by WES. Location of the p.R306C and p.G401R mutations suggests they affect Kv2.1 voltage sensing and pore gating, respectively. Indeed, the R306C mutation reduced sensitivity and cooperativity of the voltage sensor for channel opening. These effects on repetitive AP firing were so robust that firing was restricted to only one or two spikes, even in response to strong current injections. The G401R mutation nullified channel function and had a dominant-negative effect on endogenous Kv2, presumably through inhibition of pore gating. This effect caused fast dampening of repetitive firing, consistent with previous reports using another dominant-negative mutant^27^ and the Kv2 blocker GxTX^26^. Thus, both mutations significantly alter channel function of Kv2.1, and firing properties of cortical neurons, strongly suggesting they are causative for the phenotype of the two patients.

Including the two patients from this study, six patients with d*e novo KCNB1* mutations have been identified, with detailed clinical information available for four patients (excluding case 3 in Torkamani *et al.*, 2014^16^ and a patient with neurodevelopmental disorder^19^). In all these four patients, the initial symptom was developmental delay, and thereafter developed various types of infantile-onset seizures. Therefore, it might be more appropriate to state that patients with *KCNB1* mutations showed encephalopathy with seizures. Although focal seizures were also observed, generalized seizures including clonic, tonic-clonic and myoclonic seizures, and spasms were seen in common. Diffuse epileptic EEG discharges were observed in all four patients, and three of them (with p.R306C, p.G379R, and p.G401R mutations) showed spike and wave discharges. Hypotonia was observed in three patients with mutations within the pore domain (p.S347R, p.G379R, and p.G401R), but not with the p.R306C mutation. Involuntary movements and behavioural problems such as tantrums and agitation were observed in two patients (with p.G379R and p.G401R mutations). Altogether, these findings suggest that Kv2.1 dysfunction in humans is associated with diffuse brain dysfunction, manifested as developmental, neurological, or behavioural symptoms. Infantile generalized seizures with spike and wave discharges may be a key feature in suspected *KCNB1* mutations.

A study examining *Kcnb1*^−/−^ mice also reported diffuse brain dysfunction in knockout mice^28^. Approximately 10% of *Kcnb1*^−/−^ mice exhibited nonlethal, tonic-clonic seizures for several seconds during routine animal handling, and they were susceptible to chemically induced seizures. In addition, *Kcnb1*^*−/−*^ mice show hyperactive behaviour and spatial learning deficits. This suggests a critical role for Kv2.1 as a homeostatic suppressor of neuronal excitability^28^. Given there is widespread expression of Kv2.1 in the brain, including the hippocampus^11^,^12^,^13^, various generalized seizures with diffuse EEG abnormalities may originate from either the entire cortex or subcortical structures such as the hippocampus.

Although the peak amplitude of total K^+^ currents was maintained in R306C-transfected neurons, the stronger effect of R306C mutant on repetitive firing must be due to delayed activation of the R306C mutant. Delayed activation failed to produce a deep interspike voltage after the initial spike, but would have strongly inhibited subsequent spike generation through counteracting depolarizing Na^+^ entry with hyperpolarizing K^+^ efflux. This is in contrast to G401R-transfected neurons, with Na^+^ entry for the second spike generation not counteracted because of a lack of slowly activating Kv components (Fig. 4).

The difference in initial activation τ between R306C-transfected Neuro2a cells (Fig. 2E) and cortical neurons (Fig. 4C) was in part due to the use of prepulses, during which a significant fraction of R306C mutants would have been activated (Figs 3B and 4E), in cortical neurons. Thus the slowly rising component of R306C-mediated currents after the initial fast rising phase (observed in Neuro2a cells) would have mainly contributed to current time course after the prepulse in R306C-transfected neurons. Moreover, inactivation of other Kvs also shaped the time course in cortical neurons. Another possibility might be that the difference in expression level of endogenous Kv2 between Neuro2a cells and cortical neurons yielded different ratios of heteromeric combination of WT to R306C (e.g., 1:3 or 2:2) in a Kv2 tetramer and these heteromers produced somewhat different τs.

Two novel Kv2.1 mutants greatly suppressed and did not facilitate, repetitive firing in cultured cortical neurons. The mechanism by which suppression of repetitive firing leads to epileptic phenotypes in humans is currently unknown. One possibility is that suppression of firing in inhibitory GABAergic interneurons promotes epileptic discharges if Kv2.1 dominates repetitive firing in these interneurons. Nevertheless, the characteristic fast spiking of GABAergic interneurons is known to be driven mainly by Kv3/KCNC^29^,^30^, and the contribution of Kv2/KCNB to repetitive firing is expected to be minimal. Conversely, it is possible that input reduction from mutant Kv2.1-expressing pyramidal neurons to interneurons mediating feedforward and/or feedback recurrent and lateral inhibitory circuits^30^ may cause unregulated firing in the surrounding neuronal circuitry. Moreover, during seizure-like firing events, with both glutamatergic and GABAergic neuronal firing occurring excessively, intracellular Cl^−^ levels in both pyramidal neurons and interneurons can be raised^31^. This Cl^−^ accumulation may convert inhibitory GABAergic inputs into excitatory signals and promote synchronous neuronal firing throughout neuronal circuits, as demonstrated in the hippocampus^32^,^33^. Cl^−^ accumulation is mainly mediated by Cl^−^ entry through GABA_A_ receptors under depolarized conditions^31^,^34^,^35^. Cl^−^ entry would be enhanced with raised interspike voltages and/or mean membrane voltage levels during depolarizing inputs, even when repetitive spiking is suppressed as in R306C- and G401R-expressing neurons (Fig. 6). Thus, it may be possible that Kv2.1 mutations promote greater intracellular Cl^−^ accumulation during seizure-like firing events, quickly rendering GABAergic inputs excitatory and lowering the threshold for and/or prolonging the duration of epileptic synchronous firings throughout the circuits.

In summary, our data suggest that Kv2.1 functional aberrations in humans are associated with developmental delay, infantile generalized seizures, hypotonia, and behavioural problems, and also highlight a critical role for Kv2.1 in regulating neuronal firing in neuronal circuits.

## Materials and Methods

### Patients

A total of 437 patients with infantile epilepsy including Ohtahara syndrome, West syndrome, Lennox-Gastaut syndrome, or malignant migrating partial seizures of infancy were analyzed. Almost half of patients are not categorized to any existing electroclinical syndromes. Patients with mutations in known epilepsy genes including *ARX*, *KCNT1*, *KCNQ2*, *SCN1A*, *SCN2A*, *SCN8A*, *STXBP1*, *SPTAN1*, *GNAO1*, *GRIN1*, *FOXG1*, *QARS*, *EEF1A2*, *PIGA*, *CDKL5*, *SLC35A2*, *CASK*, *PCDH19* or *MECP2*, which were detected by high-resolution melting analysis, target capture analysis, direct sequencing analysis, or WES, were excluded from the study. DNA was extracted from peripheral blood leukocytes using standard methods. Clinical information and peripheral blood samples were obtained after written informed consent was provided.

### Whole exome sequencing

Genomic DNA was captured using SureSelect Human All Exon v4 or v5 (Agilent Technologies), and sequenced on an Illumina HiSeq2000 (Illumina) with 101 bp paired-end reads. Exome data processing, variant calling, and variant annotation were performed as described previously^36^. For detecting *KCNB1* mutations in WES data, we focused on rare nonsynonymous *KCNB1* variants absent in dbSNP137 (http://www.ncbi.nlm.nih.gov/projects/SNP/), Exome Variant Server database (http://evs.gs.washington.edu/EVS/), and in our in-house 575 control exomes. These *KCNB1* variants were annotated using GenBank accession number NM_004975.2. Segregation of all candidate *KCNB1* mutations was examined by Sanger sequencing using trio samples (patients and their parents). In families showing *de novo* mutations, parentage was confirmed by analyzing 12 microsatellite markers.

### Expression vector construction

A full-length human *KCNB1* cDNA clone was purchased from the Kazusa DNA Research Institute. Site-directed mutagenesis using a KOD-Plus-Mutagenesis kit (Toyobo) was performed to generate two *KCNB1* mutants: c.916C >T (p.R306C) and c.1201G >A (p.G401R). All variant cDNAs were confirmed by Sanger sequencing. WT and mutant *KCNB1* cDNAs were cloned into the pCAGGS-IRES2-nucEGFP vector^37^,^38^, to simultaneously express Kv2.1 and nuclear-localized enhanced green fluorescent protein.

### Cell preparation and plasmid transfection

The Neuro2a cell line was purchased from the European Collection of Cell Cultures (ECACC). The culture medium consisted of MEMα (with GlutaMAX, no nucleosides; Life technologies), 10% fetal bovine serum (Life technologies), and 50 U and 50 μg/ml of penicillin-streptomycin (Life technologies). Vector transfection was performed using Lipofectamine 3000 (Life technologies), according to the manufacturer’s protocol. Cells were used for experiments 2–3 days after transfection.

Primary cultured cortical neurons were prepared from the cerebral cortices of ICR mouse embryos on E_16_ as described previously^39^. Expression vectors were introduced into dissociated neurons by electroporation with an Amaxa Nucleofector device (Lonza), according to the manufacturer’s protocol. The culture medium contained Neurobasal, B27 supplement, 0.5 mM GlutaMAX-I, and 50 U and 50 μg/ml of penicillin-streptomycin (all from Life technologies). Osmolality of this culture medium was 220 mOsm/kg H_2_O. Experiments were performed after 9–14 days *in vitro*. Pyramidal-like neurons expressing nucEGFP were selected under epifluorescent illumination.

### Patch-clamp studies

Membrane currents and voltages in Neuro2a cells and primary cortical neurons were recorded using the whole-cell patch clamp technique with an EPC10 amplifier controlled via Patchmaster software (HEKA Elektronik). Records were filtered at 5 kHz and digitized at 50 kHz. Patch pipettes were fabricated from borosilicate glass capillaries using a P-97 puller (Sutter Instrument). Pipette resistance was 1.5–3 MΩ when filled with pipette solution. For voltage-clamp recordings in Neuro2a cells, the extracellular solution contained (in mM): 140 Na isethionate, 5 KCl, 2 CaCl_2_, 1 MgCl_2_, 5 Na-HEPES, 6 HEPES, 5 glucose, and 1 μM tetrodotoxin (pH 7.4, 300 mOsm/kg H_2_O). In addition, the pipette solution contained (in mM): 125 K methanesulfonate, 10 KCl, 2 MgCl_2_, 10 HEPES, 3 Na_2_ATP, 0.2 NaGTP, and 1 EGTA (pH 7.4, 280 mOsm/kg H_2_O). The liquid junction potential between these solutions was 1.4 mV. For voltage-clamp recordings in primary cortical neurons, Ca^2+^-free high-Mg^2+^ extracellular solution was used, containing (in mM): 120 Na isethionate, 5 KCl, 3 MgCl_2_, 5 Na-HEPES, 6 HEPES, 5 glucose, 0.1 EGTA, and 1 μM tetrodotoxin (pH 7.4, 260 mOsm/kg H_2_O). Pipette solution for these recordings contained (in mM): 110 K methanesulfonate, 10 KCl, 2 MgCl_2_, 10 HEPES, 3 Na_2_ATP, 0.2 NaGTP, and 1 EGTA (pH 7.4, 250 mOsm/kg H_2_O). The liquid junction potential between these solutions was 1.7 mV. Leak conductance was not subtracted from voltage-clamp recordings. For current-clamp recordings in these neurons, the extracellular solution contained (in mM): 120 NaCl, 5 KCl, 2 CaCl_2_, 1 MgCl_2_, 5 Na-HEPES, 6 HEPES, 5 glucose, 50 μM d-AP5 (2-amino-5-phosphonopentanoic acid), 10 μM CNQX (6-cyano-7-nitroquinoxaline-2,3-dione), and 50 μM picrotoxin (pH 7.4, 260 mOsm/kg H_2_O). Pipette solution for this purpose contained (in mM): 110 K methanesulfonate, 10 KCl, 2 MgCl_2_, 10 HEPES, 3 Na_2_ATP, 0.2 NaGTP, and 1 EGTA (pH 7.4, 250 mOsm/kg H_2_O). The liquid junction potential between these solutions was 5.3 mV. All liquid junction potentials were corrected online. Series resistance during recordings was maintained at < 10 MΩ and compensated for by 80%. All experiments were performed at 26–28 °C.

Activation τs of voltage-clamp Kv currents were determined by fitting the initial current rising phase from 20% of the peak rise to single or double exponentials, depending on better fit. Tail currents were elicited by a 100 ms test voltage step to −50 mV after 200 ms voltage steps ranging from −80 mV in Neuro2a cells, or from −60 mV in primary cortical neurons, to +50 mV in 10 mV increments. To produce activation curves in Figs 3B and 4E, peak amplitudes of tail currents were measured 2 ms after beginning test voltage steps, and fitted to the Boltzmann equation: I = I_base_ + I_max_/[1 + exp{(V_h_–V)/V_s_}], where I_base_ and I_max_ are the basal and maximum current amplitudes, respectively; V is the voltage level before the test voltage step (prepulse voltage); V_h_ is the half-maximal voltage; and V_s_ is the slope factor. Peak amplitudes after subtraction of I_base_ were normalized to I_max_ and mean values over the cells plotted against the prepulse voltage. To produce inactivation curves in Fig. 3D, currents were elicited by 8 s long voltage steps ranging from −100 to +30 mV in 10 mV increments, followed by a brief 1 ms pulse to +70 mV and a 100 ms test voltage step to +40 mV. The brief pulse reduced residual capacitive current components in response to the test voltage step. Peak amplitudes of current responses to test voltages steps in empty vector- and WT Kv2.1-transfected cells were fitted to the Boltzmann equation: I = I_base _+ I_max_/[1 + exp{(V–V_h_)/V_s_}], in the voltage range of long voltage steps (prepulse voltage) from −100 to 0 mV. Amplitudes were normalized to the sum of I_base_ and I_max_, and mean values over the cells plotted against the prepulse voltage. Amplitudes in the positive prepulse voltage range were slightly larger than those at 0 mV (Fig. 3D, dotted lines), indicating U-type inactivation of Kv2.1^40^,^41^,^42^. In R306C mutant-transfected cells, amplitudes were normalized to the current evoked after the prepulse to −100 mV.

Input resistance under resting conditions in current-clamp mode was measured from membrane voltage changes induced by 500 ms hyperpolarizing and depolarizing current injections in 20 or 50 pA increments from the resting membrane voltage. Resistance was determined as the linear regression slope of peak voltage changes against injected currents in the voltage range below the firing threshold and above −80 mV. Rheobase was determined by applying 500 ms depolarizing current injections in 20 pA increments from the resting membrane voltage.

### Statistics

For patch-clamp data, statistical analysis was performed using IBM SPSS ver.21 software. Data were first assessed for normality of distribution using the Kolmogorov–Smirnov test. When confirmed, multiple comparisons were performed using one-way ANOVA followed *post hoc* by Ryan–Einot–Gabriel–Welsch *F* (REGW) or Dunnett’s T3 tests, depending if equal variances could be assumed or not, respectively, from Levene statistic. When the normality was not confirmed, comparisons were performed using the Kruskal–Wallis test followed by the Dunn procedure. Data are presented as mean ± standard error of the mean.

### Study approval

Experimental protocols were approved by the institutional review board of Yokohama City University School of Medicine, and the Animal Care and Use Committee of Hamamatsu University School of Medicine. Experiments using mice conformed to the Guiding Principles for the Care and Use of Animals issued by the Physiological Society of Japan.

## Additional Information

**How to cite this article**: Saitsu, H. *et al.* *De novo KCNB1* mutations in infantile epilepsy inhibit repetitive neuronal firing. *Sci. Rep.* **5**, 15199; doi: 10.1038/srep15199 (2015).

## Supplementary Material

Supplementary Information

## Figures and Tables

**Table 1:** Clinical features of patients with *KCNB1* mutations.

	Patient 1	Patient 2	Case ID9*	Case 2*	Case 3*
**Age**	4 yr	7 yr	9 yr	7 yr	5 yr
**Sex**	Male	Male	Female	Male	Female
**Mutation**	c.1201G>A (p.G401R)	c.916C>T (p.R306C)	c.1041C>A (p.S347R)	c.1135G>A (p.G379R)	c.1121C>T (p.T374I)
**Initial symptoms**	Developmental delay	Developmental delay	Developmental delay	Developmental delay	N.D.
**Age at seizure onset**	1 yr 5 mo	1 yr	4 yr	8 mo	0 yr
**Seizure types**	Clonic at 1 yr 5 mo, focal with jerking of mouth	Spasms at 1 yr, tonic-clonic, myoclonic, and focal with head deviation at 2 yr	Tonic-clonic, tonic-atonic, focal, and focal with secondary generalization	Tonic-clonic, atonic, focal, and infantile spasms	Tonic-clonic, atypical absence, atonic, infantile spasms, and focal dyscognitive
**EEG findings**	Diffuse polyspikes and waves with intermittent multifocal spikes at 1yr 6mo	Generalized discharges of high amplitude spikes-waves and polyspikes at 2 yr 11 mo	Mild diffuse slowing and abundant bihemispheric multifocal epileptiform discharges	Hypsarrhythmia at 8 mo, diffuse polyspikes, diffuse polyspikes-waves, right temporal spikes and waves, left occipital spikes, and diffuse polyspike bursts at 5 yr	Unspecified
**Response to therapy**	Refractory	Refractory	Refractory	Refractory	Unknown
**Hypotonia**	+	−	+	+	N.D.
**Involuntary movement**	Choreic and myoclonic movement of the upper limbs	−	N.D.	Stereotyped handwringing movements	N.D.
**Intellectual disability**	Severe (no words)	Severe (no words)	+	+	Unspecified
**Motor development**	Unable to sit by 4 yr	Walking at 2 yr 6 mo	Delayed	Walking at 2 yr 6 mo	Unspecified
**MRI findings**	Normal at 9 mo, progressive brain atrophy at 1 yr 6 mo and 2 yr 2 mo	Normal at 2 yr 3 mo	Subtle volume loss in the left hippocampus	Normal at 9 mo	Normal
**Other notes**	−	Tantrum bursts, macrocephaly	Intermittent agitation, strabismus, migraine	Strabismus, tremulousness, nonverbal, in-turning of feet	Cerebral palsy

yr, years; mo, months; N.D., not described; +, present; −, absent.

^*^Torkamani A *et al.* *De novo KCNB1* mutations in epileptic encephalopathy. Ann Neurol 2014; 76: 529–40.
