# Supplementary material for: De novo KCNB1 mutations in infantile epilepsy inhibit repetitive neuronal firing
Source: Sci Rep. 2015 Oct 19;5:15199. doi: 10.1038/srep15199 (PMC4609934; doi:10.1038/srep15199)
Supplement: Supplementary Information [file srep15199-s1.doc]

**Supplementary Information**

***De novo KCNB1* mutations in infantile epilepsy inhibit repetitive neuronal firing**

Hirotomo Saitsu,^1,6^,* Tenpei Akita,^2,6^,* Jun Tohyama,^3^ Hadassa Goldberg-Stern,^4^ Yu Kobayashi,^3^ Roni Cohen,^4^ Mitsuhiro Kato,^5^ Chihiro Ohba,^1^ Satoko Miyatake,^1^ Yoshinori Tsurusaki,^1^ Mitsuko Nakashima,^1^ Noriko Miyake,^1^ Atsuo Fukuda,^2^ and Naomichi Matsumoto^1^

^1^Department of Human Genetics, Yokohama City University Graduate School of Medicine, Yokohama, Japan

^2^Department of Neurophysiology, Hamamatsu University School of Medicine, Hamamatsu, Japan

^3^Department of Pediatrics, Epilepsy Center, Nishi-Niigata Chuo National Hospital, Niigata, Japan

^4^Epilepsy Center, Schneider’s Children Medical Center, Petah Tiqwa, Israel

^5^Department of Pediatrics, Yamagata University Faculty of Medicine, Yamagata, Japan

^6^These authors contributed equally to this work.

**Case reports**

Patient 1 is a 4-year-old boy, born without asphyxia after an uneventful 40-week pregnancy to nonconsanguineous Japanese parents. At birth, his body weight was 3395 g (+0.9 s.d.), and head circumference 33.0 cm (−0.2 s.d.). His initial development was delayed as he gained social smiling, rolling over, and head control at 3, 8, and 18 months of age, respectively. At 17 months of age, he developed daily episodes of recurrent sudden bilateral upper limb jerking, upward eye deviation, vocalization, and irregular breathing, each lasting a few seconds. At 18 months of age, he showed dyskinetic movement of the upper extremities, myoclonic movement, and hypotonia without muscle weakness or spasticity. His head circumference was 45.7 cm (−1.3 s.d.). Recurrent attacks of sudden myoclonic movement of both upper limbs, upward eye deviation, and vocalization with loss of consciousness, lasting several tens of seconds and that were considered to be clonic seizures, were frequently observed. His EEG showed diffuse polyspikes and waves with intermittent multifocal spikes (Figure 7A). Ictal video-EEG recording revealed epileptic discharges corresponded to clonic seizures (Figure 7C). Brain MRI at 9 months of age showed normal results (Figure 7D), but follow-up MRI at 18 and 26 months showed progressive brain atrophy especially in the cortex (Figure 7E). His seizures were refractory to various anti-epileptic drugs including valproic acid, clobazam, zonisamide, lamotrigine nitrazepam, and levetiracetam. Adrenocorticotropic hormone aggravated his seizures. Subsequently, treatment with phenobarbital, carbamazepine, and stiripentol was started, and partially effective, although his seizures did not stop. Follow-up EEG at 3 years and 6 months showed high-amplitude (> 300 μV) polyspike and wave discharges, and independent spike discharges (Supplementary Figure S2A). He also developed focal seizure with jerking of mouth. At 4 years old, he still had weekly clonic seizures of both arms lasting tens of seconds. He achieved head control and rolling over, but spoke no meaningful words and could not sit unaided. Choreic and myoclonic movements of the upper extremities and transient eye-deviation were also observed.

Patient 2 is a 7-year-old boy, born without asphyxia by elective cesarean section after an uneventful 39-week pregnancy to nonconsanguineous parents of Ethiopian origin. His birth weight was 3400 g (+0.1 s.d.). The paternal uncle has intellectual disability of unknown etiology. A developmental delay was noted as he showed smiling at 5 months, crawling at 18 months, walking at 2 years and 6 months, and spoke no words. Seizures with flexor spasm started at 1 year of age, but he did not attract medical attention at that time. He developed generalized tonic-clonic and myoclonic seizures when he woke from sleep and during wakefulness at 2 years of age. Occasional focal seizures with head deviation were also reported. He had severe behavioural problems, manifested by tantrum bursts that were partially responsive to risperidone therapy. At 2 years and 6 months, he showed severe developmental delay and had no speech with autistic features. Neurological examination demonstrated a hyperpigmented spot on the left thigh, macrocephaly (98th percentile), normal cranial nerves, and no pyramidal or cerebellar abnormalities. Interictal EEG at 2 years and 11 months showed multiple generalized discharges of high amplitude spike-waves and polyspikes (Figure 7B). Brain MRI at 2 years and 3 months of age was normal (Figure 7F). Laboratory examination showed normal findings. Multiple anti-epileptic drugs including valproic acid, lamotrigine, clonazepam, sulthiame, levetiracetam, and phenobarbital, and a ketogenic diet, were ineffective. Interictal EEG at 5 years and 10 months showed high-amplitude polyspikes, spike-wave, and polyspike-wave discharges (Figure S2B). Currently, he is on valproic acid monotherapy with occasional myoclonic jerks and relatively well-controlled generalized tonic-clonic seizures. He has severe intellectual disability with no speech or communication. He walks independently and has special needs education.

**
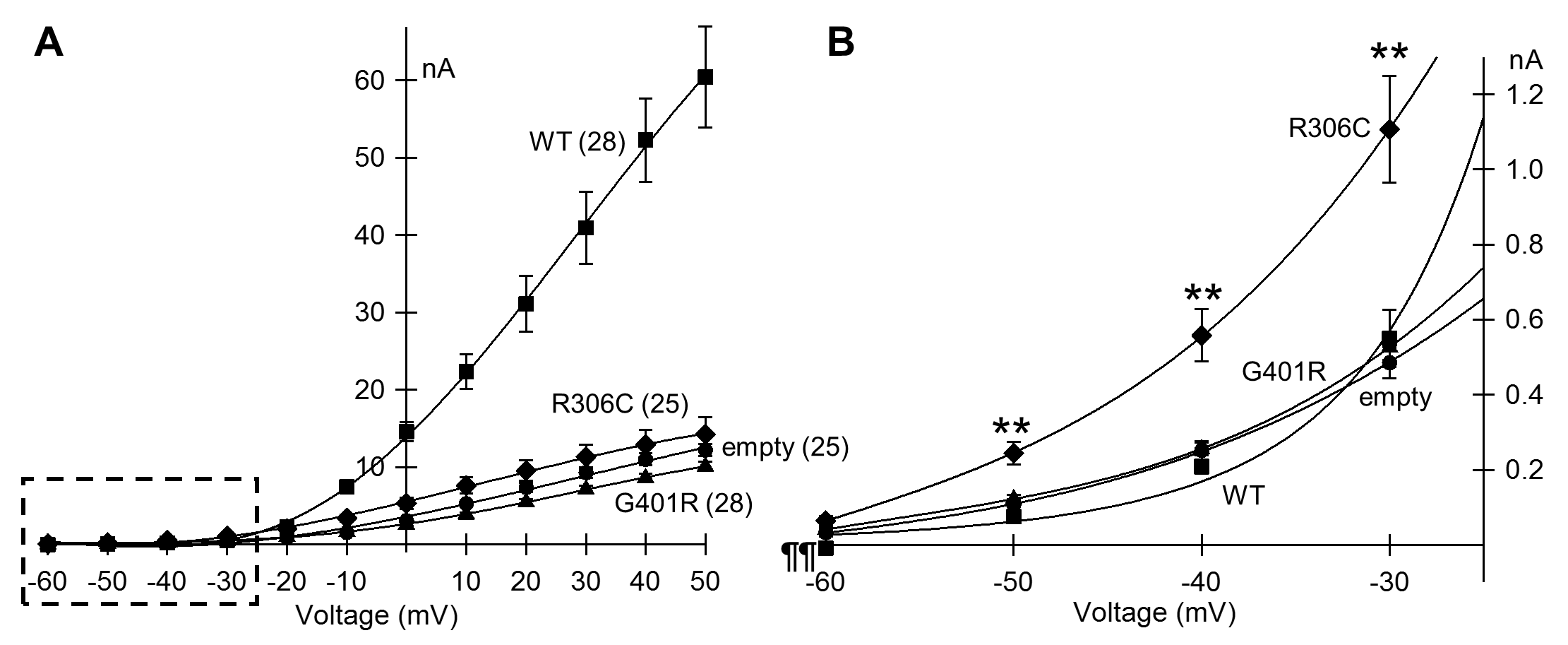
**

**Supplementary Figure S1: I-V relationships of Kv currents in the voltage range from –30 to –60 mV in Kv2.1 mutant-transfected primary cortical neurons.**

(**A**) The I-V relationships of peak currents in Fig. 4B were rescaled in absolute current level (nA). The dotted region was expanded in (**B**). (**B**) I-V relationships in the voltage range from –30 to –60 mV. In the range from –30 to –50 mV, the current in R306C (diamonds) was significantly larger (** *P* < 0.01 by Dunnett’s T3 test) than those in WT (squares), G401R (triangles) and empty (circles). At –60 mV, the current in WT was significantly smaller (¶¶ *P* < 0.01 by REGW *F* test) than those in others. The differences between the currents in G401R and empty were not significant within this voltage range.

**
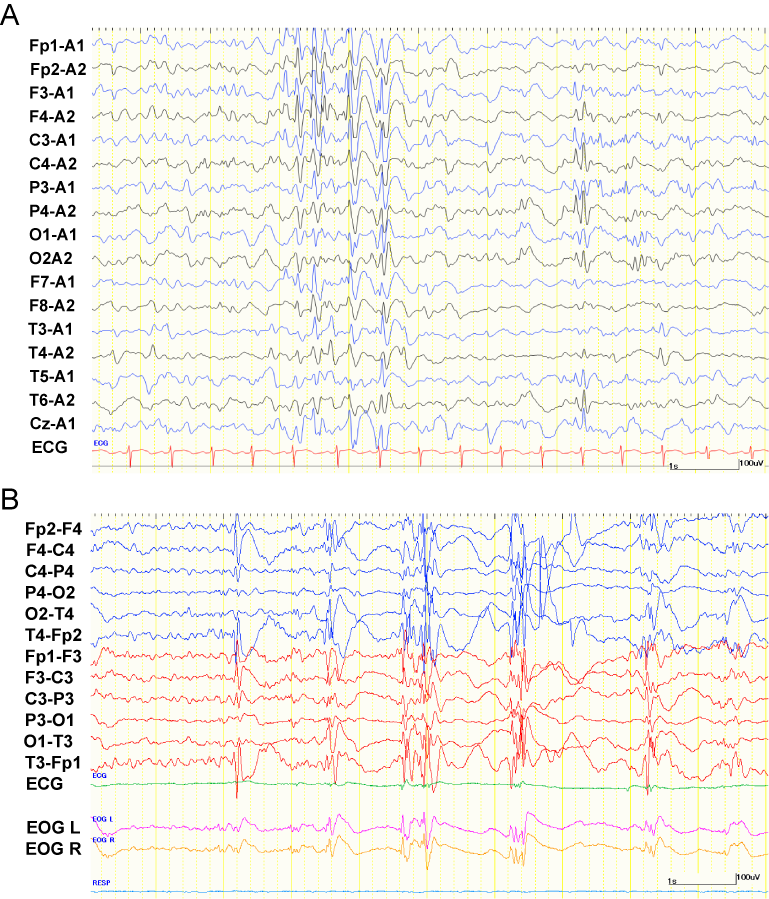
**

**Supplementary Figure S2**

(**A**) Interictal EEG of patient 1 at 3 years and 6 months showed high-amplitude polyspike-wave discharges and independent spike discharges. (**B**) Interictal EEG of patient 2 at 5 years and 10 months showed high-amplitude polyspike, spike-wave, and polyspike-wave discharges.

**Table S1. Summary of exome sequencing performance**

| Sample | Status | Origin | Mean depth^a^ | % bases above 5× | % bases above 10× | Rare variants  (filter passed)^b^ | Rare nonsynonymous variants^c^ |
| --- | --- | --- | --- | --- | --- | --- | --- |
| Patient 1 | Affected | Japanese | 138.61 | 96.1 | 94.7 | 383 | 247 |
| Father | Unaffected | Japanese | 150.91 | 96.4 | 95.6 |  |  |
| Mother | Unaffected | Japanese | 161.82 | 96.3 | 95.6 |  |  |
|  |  |  |  |  |  |  |  |
| Patient 2 | Affected | Ethiopian | 103.50 | 97.0 | 95.8 | 2165^d^ | 1204^d^ |

^a^Coverage was calculated using RefSeq gene coding sequences.

^b^Variants that passed Genome Analysis Toolkit (GATK) hand filtering, had minor allele frequencies < 1% in dbSNP135 data, and were not found in more than six of our 575 control exomes.

^c^Protein-altering and splice-affecting variants (including synonymous variants located within 2 bp of an exon-intron boundary).

^d^High numbers of rare variants were caused by ethnic differences, as our 575 control exomes consisting of Japanese individuals were used for filtering.

**Table S2. Characterization of candidate variants identified by WES**

| Patient | Chr | Gene | Mutation | Inheritance | dbSNP137 | ESP6500  data | In-house  database | SIFT | Polyphen2 | Mutation Taster |
| --- | --- | --- | --- | --- | --- | --- | --- | --- | --- | --- |
| Patient 1 | 5 | *BRD9* | c.176T>C  p.L59P | *de novo* | - | - | 0/575 | 0.00 | Possibly damaging 0.519 | Disease causing  0.9999 |
| Patient 1 | 20 | *KCNB1* | c.1201G>A  p.G401R | *de novo* | - | - | 0/575 | 0.00 | Probably damaging 1.000 | Disease causing  0.9999 |
| Patient 1 | X | *PFKFB1* | c.1259A>C  p.H420P | maternal | - | - | 1/575  (1/281 female) | 0.00 | Probably damaging 0.998 | Disease causing  0.9999 |
| Patient 1 | X | *BCORL1* | c.3346C>T  p.P1116S | maternal | - | - | 1/575  (1/281 female) | 0.50 | Benign  0.028 | Polymorphism |
|  |  |  |  |  |  |  |  |  |  |  |
| Patient 2 | 20 | *KCNB1* | c.916C>T  p.R306C | *de novo* | - | - | 0/575 | 0.00 | Probably damaging 1.000 | Disease causing  0.9999 |

SIFT (http://sift.jcvi.org/): scores < 0.05% predict intolerant substitutions.

PolyPhen-2 (http://genetics.bwh.harvard.edu/pph2/): HumVar scores are evaluated as 0.000 (most probably benign) to 1.000 (most probably damaging).

Mutation Taster (http://www.mutationtaster.org/): rapid evaluation of DNA sequence alterations. Alterations are classified as disease causing or polymorphisms.

ESP6500, 6500 exomes sequenced by the National Heart, Lung, and Blood Institute (NHLBI) Exome Sequencing Project (<http://evs.gs.washington.edu/EVS/>).
